# Modeling and Flight Experiments for Swarms of High Dynamic UAVs: A Stochastic Configuration Control System with Multiplicative Noises

**DOI:** 10.3390/s19153278

**Published:** 2019-07-25

**Authors:** Hongbo Zhao, Sentang Wu, Yongming Wen, Wenlei Liu, Xiongjun Wu

**Affiliations:** 1 School of Automation Science and Electrical Engineering, Beihang University, Beijing 100191, China; 2 Science and Technology on Information Systems Engineering Laboratory, Beijing Institute of Control & Electronics Technology, Beijing 100038, China; 3 The 802 Institute of Shanghai Academy of Space Flight Technology, The Eighth Academy of China Aerospace Science and Technology Corporation, Shanghai 200090, China

**Keywords:** stochastic system, UAV swarm, configuration control, multiplicative noises, dynamic model, stochastic robustness analysis and design

## Abstract

UAV Swarm with high dynamic configuration at a large scale requires a high-precision mathematical model to fully exploit its boundary performance. In order to instruct the engineering application with high confidence, uncertainties induced from either systematic measurement or the environment cannot be ignored. This paper investigates the $It\hat{o}$ stochastic model of the UAV Swarm system with multiplicative noises. By combining the cooperative kinematic model with a simplified individual dynamic model of fixed-wing-aircraft for the first time, the configuration control model is derived. Considering the uncertainties in actual flight, multiplicative noises are introduced to complete the $It\hat{o}$ stochastic model. Following that, the estimator and controller are designed to control the formation. The mean-square uniform boundedness condition of the proposed stochastic system is presented for the closed-loop system. In the simulation, the stochastic robustness analysis and design (SRAD) method is used to optimize the properties of the formation. More importantly, the effectiveness of the proposed model is also verified using real data of five unmanned aircrafts collected in outfield formation flight experiments.

## 1. Introduction

Swarms of UAVs, which can autonomously implement missions [1], have received significant attention in recent years. There are many application scenarios for UAV swarms, such as comprehensive combat [1], distributed reconfigurable sensor networks [2], surveillance [3], and reconnaissance systems [4]. The primary concern of the UAV swarm is the configuration control problem and related research mainly focuses on mathematical modeling [5], control strategies and methods [6,7] and collision and obstacle avoidance algorithms [8,9]. However, most of them are based on a deterministic system or a system with ideal Gaussian noises. High dynamic USCC model with multiplicative noise remains as one of the primary and practical issues for utilizing UAV swarms in engineering applications.

Generally, the stochastic differential equation refers to a stochastic process-driven system or an ordinary differential equation with a random coefficient [10,11,12,13,14]. Random factors are introduced into the system in the following three ways [10]: 1. the system’s initial conditions or inputs are taken as random variables, 2. the system’s random external disturbances, 3. the system’s parameters and structures taken as random variables. The disturbances in the UAV swarm system are mainly induced from the sensor measurement, internet transmission and the task environment, and they fall under point 2 mentioned above.

Existing work on the formation configuration control problem has been extensively investigated for low dynamic deterministic systems. In [15], the formation containment problems based on linear state equations of the multiagent systems were investigated. In [6,16], some theoretical and practical problems of multiple quadrotors were studied. In [17,18,19], the dynamical model based formation control problems of multirobot systems were studied. Most of the studies focus on low-speed vehicles, such as multiple quadrotors or multirobot systems, with an ideal environment; however, very few studies consider the formation control problems of high dynamic fixed-wing unmanned aircraft under complex environments. The mathematical modeling of stochastic high dynamic UAV swarms remains to be solved.

In this paper, we introduce the stochastic model for the following two reasons: (1) The UAV swarm, especially for high dynamic, dense configuration and large scale swarms, requires a high-precision mathematical model to describe the dynamic relationships among formation members and fully exploit its boundary performance; (2) When the UAV swarm is carrying out missions, the influence of various uncertainties (systematic measurement random interferences, network-induced random interferences and mission environment random interferences) cannot be ignored, and the relative movements of its members are usually random. Communication topology, caused by a network change, inevitably influences the process of information sending and receiving [20]. Therefore, it is necessary to combine the mathematical model of the USCC with the stochastic system to instruct the engineering practice such as cooperative detection under complex mission environments with higher confidence.

Undoubtedly, constructing a more adaptable stochastic model for multiple vehicle systems is an urgent task. The problem of Brownian motion-driven multiagent tracking was discussed in [21] and sufficient conditions for the tracking of multi-agents were obtained by using the auxiliary function of Brownian motion and random $It\hat{o}$ integral technology. A time lag multiagent system model with measurement noise was set up in [22], and the stability theory of stochastic differential equations was used. In [23], the stochastic factor has been considered in the leader-following multiagent model based on the event-triggered control strategy, *Itô* formula and stability theory. Studies [20,24,25,26,27] have considered the influence of stochastic disturbances on the multiagent system (MAS) and have established a stochastic model to control the MAS. However, existing works about the stochastic MAS are mainly based on assumed state matrices. Although the assumed formula can be applied across multiple levels, extra difficulties will occur in a certain practical system; for example, the process of combining the flight member’s dynamics model and the formation’s kinematics model is more complex; the modeling of process noises is more complex because the coefficients are presented in a very complex way in the practical system; the simulation and flight test are more difficult because of the complex system and high-risk environment. Therefore, numerous problems remain to be solved.

Typically, there are four main methods for formation coordination modeling reported in the literature: the leader-follower framework [28], virtual structure approach [29,30], behavior-based model [31,32] and graph theories [33]. Most of them focus on the consensus problems based on the kinematic model. In this paper, intending to improve both the formation properties and individual capabilities of UAV swarm in complex environments, we first use a simplified nonlinear dynamics model of the fixed-wing aircraft flight control system and then construct the group dynamic cooperative control system model together with the relative kinematic model. Based on Reynolds’ three criteria [31], the model comprehensively considers the flight member’s individual properties and the whole swarm’s cooperativeness.

To the best of our knowledge, although few efforts have been devoted to the modeling of dynamic formation systems, there is almost no literature on the stochastic model of the UAV swarms. For example, a six-degree-of-freedom (DOF) unified nonlinear dynamic model of spacecraft formation was presented in [34]; In [35,36], the formation control laws for YF-22 aircraft models with six DOF dynamics plus kinematic equations were designed. Although these formation models are efficient for the cooperative of a group without stochastic disturbance, the control strategy may be invalid when control objects are moving in the noise environment.

To the best of the authors’ knowledge, few papers discuss the quality of the configuration, and most of them have come up with an algorithm to control the formation, thereby achieving the desired configuration or avoiding collision [6,7,8]. However, they do not discuss the robustness of UAV swarms in much detail. In order to improve the robustness of the stochastic system, the parameters of the estimator and the controller are optimized by the stochastic robustness analysis and design (SRAD) method [37] in the simulation. Furthermore, few studies have carried out fixed-wing flight test experiments. A multi-UAV outfield flight experiment was carried out to verify the effectiveness of the formation collision forecast and coordination algorithm in [9]. In [36], a set of flights was performed to assess the performance of the formation control laws. To extend the previous outfield flight test results, the overall design in this paper is validated experimentally by flight testing using the leader-follower configuration.

Motivated by the discussions above, the stochastic USCC model with multiplicative noises is investigated in this paper. Compared with the existing literature, the main theoretical and experimental contributions of our work are summarized as follows:

(a) Firstly, with the aim to instruct the engineering application of UAV swarms, we construct the nonlinear formation model by combining the nonholonomic individual dynamics model of a UAV swarm with the relative cooperative kinematics model. The model comprehensively considers the personality of the individual members and the cooperation of the whole formation.
(b) Secondly, the stochastic state equation and output equation, together with the estimator and controller, finally constitute a state-feedback-based closed-loop $It\hat{o}$ stochastic system, which makes full use of the platform’s boundary performance and better matches the complex task environments to improve the cost-effectiveness of the UAV swarm system.
(c) Thirdly, most studies of formation configuration control focus on theoretical analysis, while the technology proposed in this paper is for engineering applications and has been verified by outfield flight tests.

The rest of this paper is structured as follows:

In Section 2, the problem’s formulation and preliminary studies of the USCC stochastic system are presented. In Section 3, the mathematical model of formation control stochastic system is illustrated in detail. The estimator and controller are also designed to control the formation, and SRAD has been used to optimize the controller and estimator. The mean-square uniformly bounded condition of the proposed stochastic system is then presented. In Section 4, simulations and experiments are conducted to verify the effectiveness of the model. Finally, concluding remarks are given in Section 5.

## 2. Preliminary and Problem Formulation

### 2.1. $It\hat{o}$ Stochastic System

Consider the linear $It\hat{o}$ stochastic system as the model to be investigated as follows:

(1) $$dx=\left[A(t)x+B(t)\right]dt+\sum_{i=1}^{m}\left[F_{i}(t)x+G_{i}(t)\right]dW_{i}$$

(2) $$dx=A(t)xdt+\sum_{i=1}^{m}F_{i}(t)xdW_{i}$$

where $x,B,G_{i}\in R^{n}$, $A,F_{i}\in R^{n\times n}$, $W(t)={\left[W_{1}(t),W_{2}(t),\cdots ,W_{m}(t)\right]}^{T},(t\geq 0)$ are m dimensional standard Wiener processes, which are defined in the complete probability space (Ω,F,P) and are independent of each other. Define the following matrices:

(3) $$\{\begin{array}{l} M(t)=A(t)⊕A(t)+\sum_{i=1}^{m}F_{i}(t)⊗F_{i}(t)\\ R(t)=2\left[I_{N}⊗B^{T}(t)\right]K_{N}+2\sum_{i=1}^{m}\left[I_{N}⊗G_{i}{}^{T}(t)\right]K_{N}F_{i}(t)\\ K(t)=\sum_{i=1}^{m}G_{i}(t)⊗G_{i}(t) \end{array}$$

where $N=n^{2}$, ‘⊕’ denotes the Kronecker tensor for a matrix, and $A⊕A=I_{n}⊗A+A⊗I_{n}$, ‘⊗’ is the Kronecker tensor product of a matrix.

(4) $$K_{N}=\left[\begin{array}{ccccccc} 1 & 0\cdots & 0 & 0\cdots & 0 & 0\cdots & 0\\ 0 & 0\cdots & 1 & 0\cdots & 0 & 0\cdots & 0\\ & & & \cdots \cdots & & & \\ 0 & 0\cdots & 0 & 0\cdots & 1 & 0\cdots & 0 \end{array}\right]$$

where the element ‘1’ appears in the first column, (n+1)th column and [(n−1)n+1]th column of $K_{N}$.

### 2.2. Mean-Square Uniform Boundedness of the Stochastic System

Since the stochastic system is complicated by external interferences, its stability condition is relatively strict and there is no trivial solution to the equation. To solve this problem, we take advantage of the mean-square uniform boundedness of the stochastic system. The condition for boundedness is slightly less strict than that of stability. Under the condition of boundedness, the states of the system are converging to bounded areas instead of certain stable points as time tends to infinity. Therefore, for the USCC stochastic system model, stability refers to the mean-square uniform boundedness.

**Definition 1.**  *If there is a positive number* c *:*

(5) $$\underset{t\to \infty }{\lim}\sup E\left\{{\left\|X_{ij}(t,t_{0},X_{ij0})\right\|}^{2}\right\}\leq c$$

Then the states $X_{ij}(t,t_{0},X_{ij0})$ are mean-square bounded. The subscript ‘0’ represents the initial value, $X_{ij0}$ denotes the initial states of the system which is composed of two members: i and j, $t_{0}$ denotes the initial time, t denotes the current time, ‖·‖ is the Euclidean norm, E{·} is the mathematical expectation, and sup is the minimum upper bound.

**Lemma 1.**  *[12] The necessary and sufficient condition for the mean-square boundedness of the solution for the time-varying linear stochastic system (1) is that the following time-varying linear deterministic system is bounded:*

(6) $$\dot{y}=\left[\begin{array}{cc} M(t) & R(t)\\ 0 & A(t) \end{array}\right]y+\left[\begin{array}{c} K(t)\\ B(t) \end{array}\right]$$

**Lemma 2.**  *[12] If (2) is a time-invariant linear system (i.e.,* $A,F_{i}=const,i=1,2,\cdots ,m$ *), system (2) is uniformly asymptotically stable if and only if* $M=A⊕A+\sum_{i=1}^{m}F_{i}⊗F_{i}$ *is stable, i.e.,* M *is a Hurwitz matrix, or the real parts of the eigenvalues of matrix* M *are negative.*

**Lemma 3.**  *[12] If* $B(t),G_{i}(t),(i=1,2,\cdots ,m)$ *are bounded and system (2) is mean-square uniformly asymptotically stable, then the solution of system (1) is mean-square uniformly bounded.*

Based on Lemma 1, Lemma 2, Lemma 3, we present sufficient conditions for the stability of the $It\hat{o}$ stochastic model and prove them.

**Theorem 1.**  *The sufficient conditions for the stability of the* $It\hat{o}$ *stochastic model (or the mean-square uniform boundedness of the stochastic system (1)) are:*

*(1)*  B(t) *and* $G_{i}(t)$ *are bounded.*
*(2)*  *The real part of the eigenvalues of matrix* M(t) *are negative.*

**Proof.**  For Equation (1), although we can use the conclusion of Lemma 1 to obtain the necessary and sufficient conditions for the mean-square boundedness directly, given that A and $F_{k},k=1\sim m$ are linear time-invariant matrices, we further simplify the mean-square boundedness conditions.

According to Lemma 3, if it satisfies condition (1), the mean-square uniform boundedness of the system (1) is equivalent to system (2) and is mean-square uniformly asymptotically stable:

According to Lemma 2, system (2) is mean-square uniformly asymptotically equivalent to condition (2). Proof completed. □

### 2.3. Estimator of $It\hat{o}$ Stochastic System

**Lemma 4.**  *[38] considering the* $It\hat{o}$ *stochastic system in the form as follows:*

(7) $$dx(t)=Ax(t)dt+A_{0}x(t)dw(t)+dw_{1}(t)$$

(8) $$dy(t)=Hx(t)dt+H_{0}x(t)dw(t)+dw_{2}(t)$$

*where* $x(t)\in R^{n}$ *,* $y(t)\in R^{m}$ *are system states and measured values, respectively.* A *,* $A_{0}$ *,* H *and* $H_{0}$ *are constant matrices (they can also be extended to time-varying matrices if needed).* w(t) *is a standard scalar Wiener process, as well as* $w_{1}(t)$ *and* $w_{2}(t)$ *, where* $w_{1}(t)\in R^{n}$ *and* $w_{2}(t)\in R^{m}$ *. The initial state* x(0) *is a zero mean second-order stochastic process.*

Assuming that x(0) is independent of w(t), $w_{1}(t)$, and $w_{2}(t)$, and it satisfies:

(9) $$\left\{ \begin{array}{l} E\{x(0)x^{T}(0)\}=D(0)\\ E\{dw(t)dw^{T}(t)\}=dt\\ E\{dw_{1}(t)dw_{1}{}^{T}(t)\}=Qdt\\ E\{dw_{2}(t)dw_{2}{}^{T}(t)\}=Rdt \end{array}\right.$$

Then the linear estimator with minimum mean square error is:

(10) $$d\hat{x}(t)=[A-K(t)H]\hat{x}(t)dt+K(t)dy(t)$$

(11) $$\hat{x}(0)=0$$

The gain of the estimator is:

(12) $$K(t)=[P(t)H^{T}+A_{0}D(t)H_{0}{}^{T}]{\left[H_{0}D(t)H_{0}{}^{T}+R\right]}^{-1}$$

where P(t) can be obtained as follow:

(13) $$\begin{array}{cc} dP(t)= & AP(t)dt+P(t)A^{T}dt+A_{0}D(t)A_{0}{}^{T}dt+Qdt\\ & -K(t)[H_{0}D(t)H_{0}{}^{T}+R]K^{T}(t)dt \end{array}$$

(14) P(0)=D(0)

(15) $$dD(t)=AD(t)dt+D(t)A^{T}dt+A_{0}D(t)A_{0}{}^{T}dt+Qdt$$

**Remark 1.**  *In this study, according to the above lemmas, we can construct a more applicable and effective USCC stochastic model to improve the formation properties of the UAV swarm system. Moreover, the measurement equation with Gaussian noises, the optimal estimator and controller are ingeniously involved in the closed-loop model. The mean square uniform boundedness condition of USCC stochastic system can be obtained based on the lemmas and theorem proposed above.*

## 3. USCC Stochastic System Modeling

The group dynamic cooperative control system model comprehensively considers the personality of the individual members and the cooperativeness of the whole formation based on Reynolds’ three criteria [31]. Generally, the model is built with virtual forces: individuality and interaction forces. Individuality force describes the nodes’ individual characteristics. Interaction force indicates the quality of group collaboration among nodes and describes the group dynamic cooperative characteristics, reflecting the ability to obey Reynolds’ three criteria. We will use it as a general theory to guide the modeling of the USCC stochastic system.

### 3.1. Model of Individual Flight Control System

The individual flight control system of the UAV swarm adopts the north-up-east coordinate system.

**Assumption 1.**  *The formation moves in a two-dimensional plane, thus the flight path inclination and pitch velocity are zero; the aircraft adopts side slip turning, thus the speed inclination angle, roll angle, roll angle velocity, angle of attack and side slip angle are all small values.*

**Assumption 2.**  *Thrust*P*is independent of velocity*V.

The simplified nonlinear mathematical model of individual flight control system is:

(16) $$\left\{ \begin{array}{cc} m & \frac{dV}{dt}=P-X\\ mV & \frac{d\varphi }{dt}=-P\beta +Z\\ mV & \frac{d\beta }{dt}=\omega_{y}+P\beta -Z\\ J_{y} & \frac{d\omega_{y}}{dt}=M_{y}\\ & \frac{dP}{dt}=-\frac{1}{T_{P}}P+\frac{K_{P}}{T_{P}}\delta_{Pc}\\ & \frac{d\delta_{y}}{dt}=-\frac{1}{T_{\delta_{y}}}\delta_{y}+\frac{K_{\delta_{y}}}{T_{\delta_{y}}}\delta_{yc} \end{array}\right.$$

where V is the flight velocity; φ is the flight path declination; β is the lateral slip angle; ω is the rotational angle velocity of the body’s coordinate system relative to the ground coordinate system. The subscript “y” denotes the y-component of ω. $J_{y}$ is the y-component of inertial moments of the body’s coordinate system. $M_{y}$ is the y-component of moment caused by the external force (including thrust) on the mass center; P is the thrust; X is the resistance force; Z is the lateral force; δ is the rudder declination; $K_{\delta },T_{\delta }$ are gain and time constants of the control surface response, respectively (subscripts x,y, z are aileron, rudder, and elevator, respectively); $K_{p},T_{p}$ are gain and time constants for the thrust response, respectively; $\delta_{c},\delta_{Pc}$ are rudder angle command and thrust command, respectively.

By performing a small-disturbance linearization on (16) [39], we can obtain:

(17) $$\left\{ \begin{array}{l} \frac{d\Delta V}{dt}=-\frac{X^{V}}{m}\Delta V+\frac{1}{m}\Delta P\\ \frac{d\Delta \varphi }{dt}=\frac{P-Z^{\beta }}{mV}\Delta \beta -\frac{Z^{\delta_{y}}}{mV}\Delta \delta_{y}\\ \frac{d\Delta \beta }{dt}=-\frac{P-Z^{\beta }}{mV}\Delta \beta +\Delta \omega_{y}+\frac{Z^{\delta_{y}}}{mV}\Delta \delta_{y}\\ \frac{d\Delta \omega_{y}}{dt}=(\frac{M_{y}^{\beta }}{J_{y}}-\frac{M_{y}^{\dot{\beta }}}{J_{y}}\frac{P-Z^{\beta }}{mV})\Delta \beta +(\frac{M_{y}^{\omega_{y}}}{J_{y}}+\frac{M_{y}^{\dot{\beta }}}{J_{y}})\Delta \omega_{y}+(\frac{M_{y}^{\delta_{y}}}{J_{y}}+\frac{M_{y}^{\dot{\beta }}}{J_{y}}\frac{Z^{\delta_{y}}}{mV})\Delta \delta_{y}\\ \frac{d\Delta P}{dt}=-\frac{1}{T_{P}}\Delta P+\frac{K_{P}}{T_{P}}\Delta \delta_{Pc}\\ \frac{d\Delta \delta_{y}}{dt}=-\frac{1}{T_{\delta_{y}}}\Delta \delta_{y}+\frac{K_{\delta_{y}}}{T_{\delta_{y}}}\Delta \delta_{yc} \end{array}\right.$$

where $X^{V}=\frac{\partial X}{\partial V}$, the same as other elements which is in the same form with $X^{V}$ in (17).

### 3.2. Model of Formation Control System

**Figure 1:**
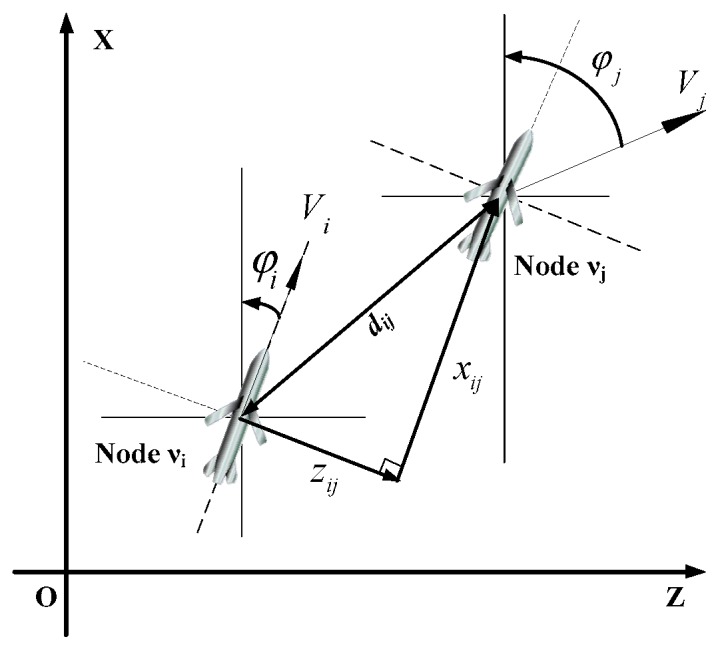
Relative movement between neighboring nodes.

For the convenience of outfield experiments and the safety of UAV swarm, we construct the model in a two-dimensional (2D) plane to make it more adaptive to complex task environments such as flat and dense formations, under which the aircraft would carry out missions at low altitude with almost no vertical maneuver space. Moreover, theoretical results can be covered fully and extended to the three-dimensional (3D) space. Therefore, we focus on the problem of USCC in the two-dimensional plane, as shown in Figure 1.

In Figure 1, $v_{i}$ and $v_{j}$ are the two nodes adjacent to each other. The flight path coordinate of node $v_{i}$ is set as the relative coordinate system, in which the x-axis represents the direction of the velocity and the z-axis is perpendicular to the x-axis shown as in Figure 1.

The ground coordinate system is set as the fixed coordinate system. $d_{ij}$ is the distance between the two nodes;$x_{ij}$ and $z_{ij}$ are the relative distances in the forward and lateral directions of the flight path coordinate system, respectively. $V_{i}$, $V_{j}$, $\varphi_{i}$ and $\varphi_{j}$ represent their velocities and flight path declinations in the ground coordinate system, respectively.

With the relationship from theoretical mechanics: absolute velocity = relative velocity + convected velocity, the following kinematics equation for node $v_{i}$ and node $v_{j}$ can be derived:

(18) $${\vec{V}}_{j}={\dot{\vec{d}}}_{ij}+{\vec{V}}_{i}+{\dot{\varphi }}_{i}\times {\vec{d}}_{ij}$$

The above equation can be decomposed in the flight path coordinate system of the node $v_{i}$ as:

(19) $$\left\{ \begin{array}{l} \frac{dx_{ij}}{dt}=V_{j}\cos(\varphi_{j}-\varphi_{i})-V_{i}+\frac{d\varphi_{i}}{dt}z_{ij}\\ \frac{dz_{ij}}{dt}=V_{j}\sin(\varphi_{j}-\varphi_{i})-\frac{d\varphi_{i}}{dt}x_{ij} \end{array}\right.$$

Substituting the second formula in (17) into (19) and performing small perturbation linearization yields:

(20) $$\left\{ \begin{array}{l} \frac{d\Delta x_{ij}}{dt}=[-1+\frac{(P_{i}-X_{i})(-P_{i}\beta_{i}+Z_{i})+m_{i}V_{i}Z_{i}{}^{V_{i}}}{m_{i}{}^{2}V_{i}{}^{2}}z_{ij}]\Delta V_{i}+V_{j}\sin(\varphi_{j}-\varphi_{i})\Delta \varphi_{i}\\ +\frac{(-P_{i}+Z^{\beta_{i}})z_{ij}}{m_{i}V_{i}}\Delta \beta_{i}-\frac{\beta_{i}z_{ij}}{m_{i}V_{i}}\Delta P_{i}-\frac{Z^{\delta_{iy}}z_{ij}}{m_{i}V_{i}}\Delta \delta_{iy}+\cos(\varphi_{j}-\varphi_{i})\Delta V_{j}-V_{j}\sin(\varphi_{j}-\varphi_{i})\Delta \varphi_{j}\\ \frac{d\Delta z_{ij}}{dt}=[\frac{(P_{i}-X_{i})(-P_{i}\beta_{i}+Z_{i})+m_{i}V_{i}Z_{i}{}^{V_{i}}}{m_{i}{}^{2}V_{i}{}^{2}}x_{ij}]\Delta V_{i}-V_{j}\cos(\varphi_{j}-\varphi_{i})\Delta \varphi_{i}\\ -\frac{(-P_{i}+Z_{i}{}^{\beta_{i}})x_{ij}}{m_{i}V_{i}}\Delta \beta_{i}+\frac{\beta_{i}x_{ij}}{m_{i}V_{i}}\Delta P_{i}+\frac{Z_{i}{}^{\delta_{i}{}_{y}}x_{ij}}{m_{i}V_{i}}\Delta \delta_{iy}+\sin(\varphi_{j}-\varphi_{i})\Delta V_{j}+V_{j}\cos(\varphi_{j}-\varphi_{i})\Delta \varphi_{j} \end{array}\right.$$

Note that the aircraft flight momentum $m_{i}V_{i}$ is relatively large. Moreover, $\beta_{i}$ has small values according to Assumption 1, and $\varphi_{j}-\varphi_{i}\approx 0$, thus $\frac{\left(P_{i}-X_{i})(-P_{i}\beta_{i}+Z_{i}\right)}{m_{i}{}^{2}V_{i}{}^{2}}\Delta V_{i}$, $\frac{\beta_{i}}{m_{i}V_{i}}\Delta P$, and $\sin(\varphi_{j}-\varphi_{i})\Delta \varphi_{i}$ are second-order small quantities. Meanwhile, $\cos(\varphi_{j}-\varphi_{i})\approx 1$, $\sin(\varphi_{j}-\varphi_{i})\approx \varphi_{j}-\varphi_{i}$. Ignoring these second-order small quantities and simplifying (20), we can get:

(21) $$\left\{ \begin{array}{l} \frac{d\Delta x_{ij}}{dt}=(\frac{Z_{i}{}^{V_{i}}z_{ij}}{m_{i}V_{i}}-1)\Delta V_{i}+\frac{(-P_{i}+Z_{i}{}^{\beta_{i}})z_{ij}}{m_{i}V_{i}}\Delta \beta_{i}-\frac{Z_{i}{}^{\delta_{iy}}z_{ij}}{m_{i}V_{i}}\Delta \delta_{iy}+\Delta V_{j}\\ \frac{d\Delta z_{ij}}{dt}=\frac{Z_{i}{}^{V_{i}}x_{ij}}{m_{i}V_{i}}\Delta V_{i}-V_{j}\Delta \varphi_{i}-\frac{(-P_{i}+Z_{i}{}^{\beta_{i}})x_{ij}}{m_{i}V_{i}}\Delta \beta_{i}+\frac{Z_{i}{}^{\delta_{i}{}_{y}}x_{ij}}{m_{i}V_{i}}\Delta \delta_{iy}+V_{j}\Delta \varphi_{j} \end{array}\right.$$

Combining Equations (17) with (19) and (21) yields the formation control system model:

(22) $$\left\{ \begin{array}{l} \frac{d\Delta x_{ij}}{dt}=a_{1}\Delta V_{i}+a_{2}\Delta \beta_{i}-a_{3}\Delta \delta_{iy}+\Delta V_{j}\\ \frac{d\Delta z_{ij}}{dt}=a_{4}\Delta V_{i}-V_{j}\Delta \varphi_{i}-a_{5}\Delta \beta_{i}+a_{6}\Delta \delta_{iy}+V_{j}\Delta \varphi_{j}\\ \frac{d\Delta V_{i}}{dt}=-a_{7}\Delta V_{i}+\frac{1}{m_{i}}\Delta P_{i}\\ \frac{d\Delta \varphi_{i}}{dt}=a_{8}\Delta \beta_{i}-a_{9}\Delta \delta_{iy}\\ \frac{d\Delta \beta_{i}}{dt}=-a_{8}\Delta \beta_{i}+\Delta \omega_{iy}+a_{9}\Delta \delta_{iy}\\ \frac{d\Delta \omega_{iy}}{dt}=a_{10}\Delta \beta_{i}+a_{11}\Delta \omega_{iy}+a_{12}\Delta \delta_{iy}\\ \frac{d\Delta P_{i}}{dt}=-\frac{1}{T_{iP}}\Delta P_{i}+\frac{K_{iP}}{T_{iP}}\Delta \delta_{iPc}\\ \frac{d\Delta \delta_{iy}}{dt}=-\frac{1}{T_{i\delta_{y}}}\Delta \delta_{iy}+\frac{K_{i\delta_{y}}}{T_{i\delta_{y}}}\Delta \delta_{iyc} \end{array}\right.$$

where $a_{1}=\frac{Z_{i}{}^{V_{i}}z_{ij}}{m_{i}V_{i}}-1$, $a_{2}=\frac{(-P_{i}+Z_{i}{}^{\beta_{i}})z_{ij}}{m_{i}V_{i}}$, $a_{3}=\frac{Z_{i}{}^{\delta_{iy}}z_{ij}}{m_{i}V_{i}}$, $a_{4}=\frac{Z_{i}{}^{V_{i}}x_{ij}}{m_{i}V_{i}}$, $a_{5}=\frac{(-P_{i}+Z_{i}{}^{\beta_{i}})x_{ij}}{m_{i}V_{i}}$, $a_{6}=\frac{Z_{i}{}^{\delta_{i}{}_{y}}x_{ij}}{m_{i}V_{i}}$, $a_{7}=\frac{X_{i}{}^{V_{i}}}{m_{i}}$, $a_{8}=\frac{P_{i}-Z_{i}{}^{\beta_{i}}}{m_{i}V_{i}}$, $a_{9}=\frac{Z_{i}{}^{\delta_{iy}}}{m_{i}V_{i}}$, $a_{10}=\frac{M_{iy}^{\beta_{i}}}{J_{iy}}-\frac{M_{iy}^{{\dot{\beta }}_{i}}}{J_{iy}}\frac{P_{i}-Z_{i}{}^{\beta_{i}}}{m_{i}V_{i}}$, $a_{11}=\frac{M_{iy}^{\omega_{iy}}}{J_{iy}}+\frac{M_{iy}^{{\dot{\beta }}_{i}}}{J_{iy}}$, $a_{12}=\frac{M_{iy}^{\delta_{iy}}}{J_{iy}}+\frac{M_{iy}^{{\dot{\beta }}_{i}}}{J_{iy}}\frac{Z_{i}{}^{\delta_{iy}}}{m_{i}V_{i}}$.

Note that: the state coefficients $V_{i}$, $P_{i}$, $x_{ij}$, $z_{ij}$ and $Z_{i}{}^{V_{i}}$ in (22) are obtained at the balanced point.

### 3.3. Random Noises Analysis and Its Modeling

#### 3.3.1. Process Noises

The formation could be easily affected by various forces in the atmosphere that cannot be accurately measured in advance. Therefore, the process noises cannot be ignored.

For the node $\nu_{i}$, assuming that the mass $m_{i}$, velocity $V_{j}$ and flight path declination angle $\varphi_{j}$ of the adjacent node $\nu_{j}$, which are obtained from the supporting network, are given values (i.e., consider that $V_{j}$ and $\varphi_{j}$ are already estimated in $\nu_{j}$, and ignore the random transmission interference), but $x_{ij}$, $z_{ij}$, $V_{i}$, $P_{i}$, $X_{i}{}^{V_{i}}$, $Z_{i}{}^{V_{i}}$, $Z_{i}{}^{\beta_{i}}$, $Z_{i}{}^{\delta_{iy}}$, $M_{iy}^{\beta_{i}}$, $M_{iy}^{{\dot{\beta }}_{i}}$, $M_{iy}^{\omega_{iy}}$ and $M_{iy}^{\delta_{iy}}$ are determined by the aircraft’s instantaneous state (such as velocity, altitude, attack angle, yaw angle, etc.); These states and their influences are random in the real flight environment. Therefore, based on the central limit theorem, we assume that the above parameters approximately obey the Gaussian distribution, that is:

(23) $$\begin{array}{llll} (1)\left\{ \begin{array}{l} x_{ij}=x_{ijb}+w_{x_{ij}}\\ w_{x_{ij}}\sim N(0,\sigma_{x_{ij}}^{2}) \end{array}\right., & (2)\left\{ \begin{array}{l} z_{ij}=z_{ijb}+w_{z_{ij}}\\ w_{z_{ij}}∼N(0,\sigma_{z_{ij}}^{2}) \end{array}\right., & (3)\left\{ \begin{array}{l} V_{i}=V_{ib}+w_{V_{i}}\\ w_{V_{i}}∼N(0,\sigma_{V_{i}}^{2}) \end{array}\right., & (4)\left\{ \begin{array}{l} P_{i}=P_{ib}+w_{P_{i}}\\ w_{P_{i}}∼N(0,\sigma_{P_{i}}^{2}) \end{array}\right.,\\ (5)\left\{ \begin{array}{l} X_{i}{}^{V_{i}}=X_{ib}{}^{V_{i}}+w_{X_{i}{}^{V_{i}}}\\ w_{X_{i}{}^{V_{i}}}\sim N(0,\sigma_{X_{i}{}^{V_{i}}}^{2}) \end{array}\right., & (6)\left\{ \begin{array}{l} Z_{i}{}^{V_{i}}=Z_{ib}{}^{V_{i}}+w_{Z_{i}{}^{V_{i}}}\\ w_{Z_{i}{}^{V_{i}}}∼N(0,\sigma_{Z_{i}{}^{V_{i}}}^{2}) \end{array}\right., & (7)\left\{ \begin{array}{l} Z_{i}{}^{\beta_{i}}=Z_{ib}{}^{\beta_{i}}+w_{Z_{i}{}^{\beta_{i}}}\\ w_{Z_{i}{}^{\beta_{i}}}∼N(0,\sigma_{Z_{i}{}^{\beta_{i}}}^{2}) \end{array}\right., & (8)\left\{ \begin{array}{l} Z_{i}{}^{\delta_{i}{}_{y}}=Z_{ib}{}^{\delta_{i}{}_{y}}+w_{Z_{i}{}^{\delta_{i}{}_{y}}}\\ w_{Z_{i}{}^{\delta_{i}{}_{y}}}∼N(0,\sigma_{Z_{i}{}^{\delta_{i}{}_{y}}}^{2}) \end{array}\right.,\\ (9)\left\{ \begin{array}{l} M_{iy}^{\beta_{i}}=M_{iyb}^{\beta_{i}}+w_{M_{iy}^{\beta_{i}}}\\ w_{M_{iy}^{\beta_{i}}}\sim N(0,\sigma_{M_{iy}^{\beta_{i}}}^{2}) \end{array}\right., & (10)\left\{ \begin{array}{l} M_{iy}^{{\dot{\beta }}_{i}}=M_{iyb}^{{\dot{\beta }}_{i}}+w_{M_{iy}^{{\dot{\beta }}_{i}}}\\ w_{M_{iy}^{{\dot{\beta }}_{i}}}∼N(0,\sigma_{M_{iy}^{{\dot{\beta }}_{i}}}^{2}) \end{array}\right., & (11)\left\{ \begin{array}{l} M_{iy}^{\omega_{iy}}=M_{iyb}^{\omega_{iy}}+w_{M_{iy}^{\omega_{iy}}}\\ w_{M_{iy}^{\omega_{iy}}}∼N(0,\sigma_{M_{iy}^{\omega_{iy}}}^{2}) \end{array}\right., & (12)\left\{ \begin{array}{l} M_{iy}^{\delta_{iy}}=M_{iyb}^{\delta_{iy}}+w_{M_{iy}^{\delta_{iy}}}\\ w_{M_{iy}^{\delta_{iy}}}∼N(0,\sigma_{M_{iy}^{\delta_{iy}}}^{2}) \end{array}\right.. \end{array}$$

The subscript “b” denotes that the values are determined and they are obtained at the balanced point. The formation states do not change much when they fly around the balanced point; thus, the variances of the random variables are approximately constant, and it can be assumed that the above parameters are independent of each other. In the following, we use $n_{1},n_{2},\cdots ,n_{12}$ to represent the random variables in (23).

For $a_{1}=\frac{Z_{i}{}^{V_{i}}z_{ij}}{m_{i}V_{i}}-1$, substituting (2) (3) (6) in (23) into $a_{1}$ yields:

(24) $$a_{1}=\frac{Z_{i}{}^{V_{i}}z_{ij}}{m_{i}V_{i}}-1=\frac{\left(Z_{ib}{}^{V_{i}}+w_{Z_{i}{}^{V_{i}}})(z_{ijb}+w_{z_{ij}}\right)}{m_{i}(V_{ib}+w_{V_{i}})}-1=\frac{Z_{ib}{}^{V_{i}}z_{ijb}}{m_{i}(V_{ib}+w_{V_{i}})}-1+\frac{Z_{ib}{}^{V_{i}}w_{z_{ij}}}{m_{i}(V_{ib}+w_{V_{i}})}+\frac{z_{ijb}w_{Z_{i}{}^{V_{i}}}}{m_{i}(V_{ib}+w_{V_{i}})}+\frac{w_{Z_{i}{}^{V_{i}}}w_{z_{ij}}}{m_{i}(V_{ib}+w_{V_{i}})}$$

Assume that $w_{V_{i}}$ is relatively small compared to the aircraft speed $V_{ib}$. Since $V_{ib}+w_{V_{i}}$ is in the denominator, the impact of $w_{V_{i}}$ on $a_{1}$ is small and can be ignored; assuming that both $w_{Z_{i}{}^{V_{i}}}$ and $w_{z_{ij}}$ are small,$w_{Z_{i}{}^{V_{i}}}w_{z_{ij}}$ is a second-order small quantity and can be ignored. Then we get:

(25) $$a_{1}=(\frac{Z_{ib}{}^{V_{i}}z_{ijb}}{m_{i}V_{ib}}-1)+\frac{z_{ijb}w_{Z_{i}{}^{V_{i}}}}{m_{i}V_{ib}}+\frac{Z_{ib}{}^{V_{i}}w_{z_{ij}}}{m_{i}V_{ib}}=(\frac{Z_{ib}{}^{V_{i}}z_{ijb}}{m_{i}V_{ib}}-1)+\frac{Z_{ib}{}^{V_{i}}\sigma_{z_{ij}}}{m_{i}V_{ib}}n_{2}+\frac{z_{ijb}\sigma_{Z_{i}{}^{V_{i}}}}{m_{i}V_{ib}}n_{6}≜a_{1b}+a_{1b2}n_{2}+a_{1b6}n_{6}$$

Assuming that all the random parts of (23) are small, and ignoring the second-order small quantity. For the same reason as $a_{1}$, the expression of the coefficients $a_{2}∼a_{15}$ could be derived. The results are given as follows:

(26) $$a_{2}=\frac{(-P_{ib}+Z_{ib}{}^{\beta_{i}})z_{ijb}}{m_{i}V_{ib}}+\frac{(-P_{ib}+Z_{ib}{}^{\beta_{i}})\sigma_{z_{ij}}}{m_{i}V_{ib}}n_{2}+\frac{-z_{ijb}\sigma_{P_{i}}}{m_{i}V_{ib}}n_{4}+\frac{z_{ijb}\sigma_{Z_{i}{}^{\beta_{i}}}}{m_{i}V_{ib}}n_{7}≜a_{2b}+a_{2b2}n_{2}+a_{2b4}n_{4}+a_{2b7}n_{7}$$

(27) $$a_{3}=\frac{Z_{ib}{}^{\delta_{iy}}z_{ijb}}{m_{i}V_{ib}}+\frac{Z_{ib}{}^{\delta_{iy}}\sigma_{z_{ij}}}{m_{i}V_{ib}}n_{2}+\frac{z_{ijb}\sigma_{Z_{i}{}^{\delta_{iy}}}}{m_{i}V_{ib}}n_{8}≜a_{3b}+a_{3b2}n_{2}+a_{3b8}n_{8}$$

(28) $$a_{4}=\frac{Z_{ib}{}^{V_{i}}x_{ijb}}{m_{i}V_{ib}}+\frac{Z_{ib}{}^{V_{i}}\sigma_{x_{ij}}}{m_{i}V_{ib}}n_{1}+\frac{x_{ijb}\sigma_{Z_{i}{}^{V_{i}}}}{m_{i}V_{ib}}n_{6}≜a_{4b}+a_{4b1}n_{1}+a_{4b6}n_{6}$$

(29) $$a_{5}=\frac{(-P_{ib}+Z_{ib}{}^{\beta_{i}})x_{ijb}}{m_{i}V_{ib}}+\frac{(-P_{ib}+Z_{ib}{}^{\beta_{i}})\sigma_{x_{ij}}}{m_{i}V_{ib}}n_{1}+\frac{-x_{ijb}\sigma_{P_{i}}}{m_{i}V_{ib}}n_{4}+\frac{x_{ijb}\sigma_{Z_{i}{}^{\beta_{i}}}}{m_{i}V_{ib}}n_{7}≜a_{5b}+a_{5b1}n_{1}+a_{5b4}n_{4}+a_{5b7}n_{7}$$

(30) $$a_{6}=\frac{Z_{ib}{}^{\delta_{i}{}_{y}}x_{ijb}}{m_{i}V_{ib}}+\frac{Z_{ib}{}^{\delta_{i}{}_{y}}\sigma_{x_{ij}}}{m_{i}V_{ib}}n_{1}+\frac{x_{ijb}\sigma_{Z_{i}{}^{\delta_{i}{}_{y}}}}{m_{i}V_{ib}}n_{8}≜a_{6b}+a_{6b1}n_{1}+a_{6b8}n_{8}$$

(31) $$a_{7}=\frac{X_{ib}{}^{V_{i}}}{m_{i}}+\frac{\sigma_{X_{i}{}^{V_{i}}}}{m_{i}}n_{5}≜a_{7b}+a_{7b5}n_{5}$$

(32) $$a_{8}=\frac{P_{ib}-Z_{ib}{}^{\beta_{i}}}{m_{i}V_{ib}}+\frac{\sigma_{P_{i}}}{m_{i}V_{ib}}n_{4}+\frac{-\sigma_{Z_{i}{}^{\beta_{i}}}}{m_{i}V_{ib}}n_{7}≜a_{8b}+a_{8b4}n_{4}+a_{8b7}n_{7}$$

(33) $$a_{9}=\frac{Z_{ib}{}^{\delta_{iy}}}{m_{i}V_{ib}}+\frac{\sigma_{Z_{i}{}^{\delta_{iy}}}}{m_{i}V_{ib}}n_{8}≜a_{9b}+a_{9b8}n_{8}$$

(34) $$\begin{array}{cc} a_{10} & =(\frac{M_{iyb}^{\beta_{i}}}{J_{iy}}-\frac{M_{iyb}^{{\dot{\beta }}_{i}}}{J_{iy}}\frac{P_{ib}-Z_{ib}{}^{\beta_{i}}}{m_{i}V_{ib}})+\frac{-M_{iyb}^{{\dot{\beta }}_{i}}\sigma_{P_{i}}}{J_{iy}m_{i}V_{ib}}n_{4}+\frac{M_{iyb}^{{\dot{\beta }}_{i}}\sigma_{Z_{i}{}^{\beta_{i}}}}{J_{iy}m_{i}V_{ib}}n_{7}+\frac{\sigma_{M_{iy}^{\beta_{i}}}}{J_{iy}}n_{9}+\frac{-(P_{ib}-Z_{ib}{}^{\beta_{i}})\sigma_{M_{iy}^{{\dot{\beta }}_{i}}}}{J_{iy}m_{i}V_{ib}}n_{10}\\ & ≜a_{10b}+a_{10b4}n_{4}+a_{10b7}n_{7}+a_{10b9}n_{9}+a_{10b10}n_{10} \end{array}$$

(35) $$a_{11}=\frac{M_{iyb}^{\omega_{iy}}+M_{iyb}^{{\dot{\beta }}_{i}}}{J_{iy}}+\frac{\sigma_{M_{iy}^{{\dot{\beta }}_{i}}}}{J_{iy}}n_{10}+\frac{\sigma_{M_{iy}^{\omega_{iy}}}}{J_{iy}}n_{11}≜a_{11b}+a_{11b10}n_{10}+a_{11b11}n_{11}$$

(36) $$\begin{array}{cc} a_{12}= & (\frac{M_{iyb}^{\delta_{iy}}}{J_{iy}}+\frac{M_{iyb}^{{\dot{\beta }}_{i}}}{J_{iy}}\frac{Z_{ib}{}^{\delta_{iy}}}{m_{i}V_{ib}})+\frac{M_{iyb}^{{\dot{\beta }}_{i}}\sigma_{Z_{i}{}^{\delta_{iy}}}}{J_{iy}m_{i}V_{ib}}n_{8}+\frac{Z_{ib}{}^{\delta_{iy}}\sigma_{M_{iy}^{{\dot{\beta }}_{i}}}}{J_{iy}m_{i}V_{ib}}n_{10}+\frac{\sigma_{M_{iy}^{\delta_{iy}}}}{J_{iy}}n_{12}\\ & ≜a_{12b}+a_{12b8}n_{8}+a_{12b10}n_{10}+a_{12b12}n_{12} \end{array}$$

The states of the system are $X_{ij}={\left[\begin{array}{cccccccc} \Delta x_{ij} & \Delta z_{ij} & \Delta V_{i} & \Delta \varphi_{i} & \Delta \beta_{i} & \Delta \omega_{iy} & \Delta P_{i} & \Delta \delta_{iy} \end{array}\right]}^{T}$; the inputs are $U_{ij}={\left[\begin{array}{cccc} \Delta \delta_{iPc} & \Delta \delta_{iyc} & \Delta V_{j} & \Delta \varphi_{j} \end{array}\right]}^{T}$; $\Delta V_{j}$ and $\Delta \varphi_{j}$ are determined random inputs. Substituting (25) to (36) into (22), then the open-loop state equation of the formation stochastic system could be get:

(37) $${\dot{X}}_{ij}=A_{ij}X_{ij}+B_{ij}U_{ij}+\sum_{k=1}^{12}F_{ijk}X_{ij}n_{k}$$

where $A_{ij}=\left[\begin{array}{cccccccc} 0 & 0 & a_{1b} & 0 & a_{2b} & 0 & 0 & -a_{3b}\\ 0 & 0 & a_{4b} & -V_{j} & -a_{5b} & 0 & 0 & a_{6b}\\ 0 & 0 & -a_{7b} & 0 & 0 & 0 & 1/m_{i} & 0\\ 0 & 0 & 0 & 0 & a_{8b} & 0 & 0 & -a_{9b}\\ 0 & 0 & 0 & 0 & -a_{8b} & 1 & 0 & a_{9b}\\ 0 & 0 & 0 & 0 & a_{10b} & a_{11b} & 0 & a_{12b}\\ 0 & 0 & 0 & 0 & 0 & 0 & -1/T_{iP} & 0\\ 0 & 0 & 0 & 0 & 0 & 0 & 0 & -1/T_{i\delta_{y}} \end{array}\right],$ $B_{ij}=\left[\begin{array}{cccc} 0 & 0 & 1 & 0\\ 0 & 0 & 0 & V_{j}\\ 0 & 0 & 0 & 0\\ 0 & 0 & 0 & 0\\ 0 & 0 & 0 & 0\\ 0 & 0 & 0 & 0\\ K_{iP}/T_{iP} & 0 & 0 & 0\\ 0 & K_{i\delta_{y}}/T_{i\delta_{y}} & 0 & 0 \end{array}\right].$ For convenience of description, we define the square matrix $T_{mn}$ whose order is eight and has only one nonzero element; the value of the nonzero element is ‘1’ and it lies in column m and row *n*.

Then matrices $F_{ijk}(k=1,2,\ldots ,12)$ can be described as follows:

$$\begin{array}{l} F_{ij1}=a_{4b1}T_{23}-a_{6b1}T_{25}+a_{7b1}T_{28},\\ F_{ij2}=a_{2b2}T_{15}-a_{3b2}T_{18},\\ F_{ij3}=a_{1b3}T_{13},\\ F_{ij4}=a_{2b4}T_{15}-a_{5b4}T_{25}+a_{8b4}T_{45}-a_{89b4}T_{55}+a_{10b4}T_{65},\\ F_{ij5}=-a_{7b5}T_{33},\\ F_{ij6}=a_{1b6}T_{13}+a_{4b6}T_{23},\\ F_{ij7}=a_{2b7}T_{15}-a_{5b7}T_{25}+a_{8b7}T_{45}-a_{8b7}T_{55}+a_{10b7}T_{65},\\ F_{ij8}=-a_{3b8}T_{18}+a_{6b8}T_{28}-a_{9b8}T_{48}+a_{9b8}T_{58}+a_{12b8}T_{68},\\ F_{ij9}=a_{10b9}T_{65},\\ F_{ij10}=a_{10b10}T_{65}+a_{11b10}T_{66}+a_{12b10}T_{68},\\ F_{ij11}=a_{11b11}T_{66},\\ F_{ij12}=a_{12b12}T_{68}. \end{array}$$

#### 3.3.2. Measurement Noises

The states of the stochastic system (37) are measured by the support network and relative navigation in the UAV swarm’s information acquisition system. The measuring vector is defined as: $Y_{ij}={\left[\begin{array}{ccccccc} \Delta x_{ijm} & \Delta z_{ijm} & \Delta V_{im} & \Delta \varphi_{im} & \Delta \beta_{im} & \Delta \omega_{iym} & \Delta \delta_{iym} \end{array}\right]}^{T}$, assuming that $\Delta P_{i}$ cannot be measured. These measured values are mainly affected by sensor measurement, network transmission and random disturbances in the flight environment. Assuming that the measured noises of the system approximately obey the Gaussian distribution, whose mathematical expectation is 0 and variance is $\sigma_{m}{}^{2}$, the measurement equation is:

(38) $$\left\{ \begin{array}{l} \Delta x_{ijm}=\Delta x_{ij}+\sigma_{\Delta x_{ijm}}n_{13}\\ \Delta z_{ijm}=\Delta z_{ij}+\sigma_{\Delta z_{ijm}}n_{14}\\ \Delta V_{im}=\Delta V_{i}+\sigma_{\Delta v_{im}}n_{15}\\ \Delta \varphi_{im}=\Delta \varphi_{i}+\sigma_{\Delta \varphi_{im}}n_{16}\\ \Delta \beta_{im}=\Delta \beta_{i}+\sigma_{\Delta \beta_{im}}n_{17}\\ \Delta \omega_{iym}=\Delta \omega_{iy}+\sigma_{\Delta \omega_{iym}}n_{18}\\ \Delta \delta_{iym}=\Delta \delta_{iy}+\sigma_{\Delta \delta_{iym}}n_{19} \end{array}\right.$$

The subscript “m” means the measured value of the system, $n_{13},n_{15},\cdots ,n_{19}$ are standard Gaussian white noises independent of each other. They are also independent of $n_{1},n_{2},\cdots ,n_{12}$.

In summary, the measurement equation is:

(39) $$Y_{ij}=H_{ij}X_{ij}+\sum_{k=13}^{19}E_{ijk}n_{k}$$

where, $H_{ij}=\left[\begin{array}{cccccccc} 1 & 0 & & & & & & \\ & 1 & & & & & & \\ & & 1 & & & & & \\ & & & 1 & \ddots & & & \\ & & & & 1 & & & \\ & & & & & 1 & 0 & \\ & & & & & & 0 & 1 \end{array}\right]$, $E_{ij13}=\left[\begin{array}{c} \sigma_{\Delta x_{ijm}}\\ 0\\ 0\\ 0\\ 0\\ 0\\ 0 \end{array}\right]$, $E_{ij14}=\left[\begin{array}{c} 0\\ \sigma_{\Delta z_{ijm}}\\ 0\\ 0\\ 0\\ 0\\ 0 \end{array}\right]$, $E_{ij15}=\left[\begin{array}{c} 0\\ 0\\ \sigma_{\Delta v_{im}}\\ 0\\ 0\\ 0\\ 0 \end{array}\right]$, $E_{ij16}=\left[\begin{array}{c} 0\\ 0\\ 0\\ \sigma_{\Delta \varphi_{im}}\\ 0\\ 0\\ 0 \end{array}\right]$, $E_{ij17}=\left[\begin{array}{c} 0\\ 0\\ 0\\ 0\\ \sigma_{\Delta \beta_{im}}\\ 0\\ 0 \end{array}\right]$, $E_{ij18}=\left[\begin{array}{c} 0\\ 0\\ 0\\ 0\\ 0\\ \sigma_{\Delta \omega_{iym}}\\ 0 \end{array}\right]$, $E_{ij19}=\left[\begin{array}{c} 0\\ 0\\ 0\\ 0\\ 0\\ 0\\ \sigma_{\Delta \delta_{iym}} \end{array}\right]$.

### 3.4. Formation Estimator Design

For the state estimating problem of the formation control stochastic system (37) and (39), we use a novel $It\hat{o}$ stochastic system estimator which has a fixed gain according to [38].

In Lemma 4, the gain of the estimator is time-varying, but in this paper, the USCC problem is investigated during cruising and the formation maintains a certain configuration with certain speed and height in that period, the formation does not change very much. Therefore, we use an estimator with fixed gain in (10) to estimate states, which can significantly simplify the computation and improve the real-time performance of the system.

The formation control stochastic system (37) and (39) can be described as the following $It\hat{o}$ stochastic system:

(40) $$dX_{ij}(t)=A_{ij}X_{ij}(t)dt+B_{ij}U_{ij}(t)dt+\sum_{k=1}^{12}F_{ijk}X_{ij}(t)dW_{k}(t)$$

(41) $$dY_{ij}(t)=H_{ij}dX_{ij}(t)+\sum_{k=13}^{19}E_{ijk}dW_{k}(t)$$

where $W_{k}(t)$ (k=1~12) in (40) are standard scalar Wiener processes. $\sum_{k=13}^{19}E_{ijk}dW_{k}(t)$ is a 7-dimension Wiener process.

The initial states $X_{ij}(0)$ satisfy:

(42) $$\left\{ \begin{array}{l} E\{X_{ij}(0)X_{ij}{}^{T}(0)\}=D_{ij}(0)\\ E\{dW_{k}(t)dW_{k}{}^{T}(t)\}=dt\\ E\{[\sum_{k=13}^{19}E_{ijk}dW_{k}(t)][\sum_{k=13}^{19}E_{ijk}dW_{k}(t){]}^{T}\}=R_{ij}dt \end{array}\right.$$

Then, substituting (40) into (41) and the fixed gain estimator of the formation control stochastic system can be obtained by Lemma 4:

(43) $$d{\hat{X}}_{ij}(t)=(A_{ij}-K_{f}H_{ij}A_{ij}){\hat{X}}_{ij}(t)dt+B_{ij}U_{ij}(t)dt+K_{f}dY_{ij}(t)$$

(44) $${\hat{X}}_{ij}(0)=0$$

where ${\hat{X}}_{ij}={\left[\begin{array}{cccccccc} \Delta {\hat{x}}_{ij} & \Delta {\hat{z}}_{ij} & \Delta {\hat{V}}_{i} & \Delta {\hat{\varphi }}_{i} & \Delta {\hat{\beta }}_{i} & \Delta {\hat{\omega }}_{iy} & \Delta {\hat{P}}_{i} & \Delta {\hat{\delta }}_{iy} \end{array}\right]}^{T}$ is the state estimate vector; $K_{f}$ is fixed gain of the estimator. $U_{ij}$ is the control input.

### 3.5. Formation Controller Design

It can be seen from (22) that the system states have a high degree of coupling between each other (such as the forward distance $\Delta x_{ij}$ and the sideslip angle $\Delta \beta_{i}$, the lateral distance $\Delta z_{ij}$ and $\Delta V_{i}$, they all have high degree of coupling between each other), so the forward and lateral distance will be controlled with a couple in this paper. The PID-based formation control system structure is a commonly used design method in the engineering application at present [40]. According to the clustering algorithm proposed in [41], the PID formation controller we adopted is:

(45) $$U_{ij}=K_{c}K_{\omega ij}({\hat{X}}_{ij}-X_{ij}^{*})+U_{jd}$$

(46) $$X_{ij}^{*}={\left[\Delta x_{ij}^{*},\Delta z_{ij}^{*},\Delta V_{i}^{*},\Delta \varphi_{i}^{*},\Delta \beta_{i}^{*},0,0,0\right]}^{T}$$

(47) $$K_{\omega ij}=diag(\omega_{ij},\omega_{ij},1,1,1,1,1,1)$$

(48) $$U_{jd}={\left[0,0,\Delta V_{j},\Delta \varphi_{j}\right]}^{T}$$

where ${\hat{X}}_{ij}$ is the output of the estimator; $X_{ij}^{*}$ is the system command; superscript “∗” indicates the command, the same as below; $U_{jd}$ is the determined interference input vector of the adjacent node; $K_{c}\in R^{4\times 8}$ is the control law, in which the last two rows in $K_{c}$ are zero vectors because $\Delta V_{j}$ and $\Delta \varphi_{j}$ are the determined interference inputs in $U_{ij}$; $K_{\omega ij}$ is the adjacency adjustment matrix, $0\leq \omega_{ij}\leq 1$ are adjacency coefficients. The larger it is, the stronger the adjacency relationship between node $\nu_{i}$ and $\nu_{j}$.

The key to designing a better PID controller is to contrive the proper PID gain parameters. In order to get a better parameter of the feedback coefficients, we use the SRAD method which combines the genetic algorithm and Monte Carlo simulation to improve the robustness of the stochastic system.

We can see from (45) that $K_{c\Delta v_{i}}(\Delta {\hat{V}}_{i}-\Delta V_{i}^{*})$ and $K_{c\Delta \varphi_{i}}(\Delta {\hat{\varphi }}_{i}-\Delta \varphi_{i}^{*})$ reflect the individuality forces of the individual aircraft, $K_{c\Delta x_{ij}}\omega_{ij}(\Delta {\hat{x}}_{ij}-\Delta x_{ij}^{*})$ and $K_{c\Delta z_{ij}}\omega_{ij}(\Delta {\hat{z}}_{ij}-\Delta z_{ij}^{*})$ reflecting the interaction forces which represent the quality of the formation cooperation. Both of them can contribute to maintaining the configuration during formation maneuvering.

### 3.6. Closed-Loop USCC Stochastic System

**Figure 2:**
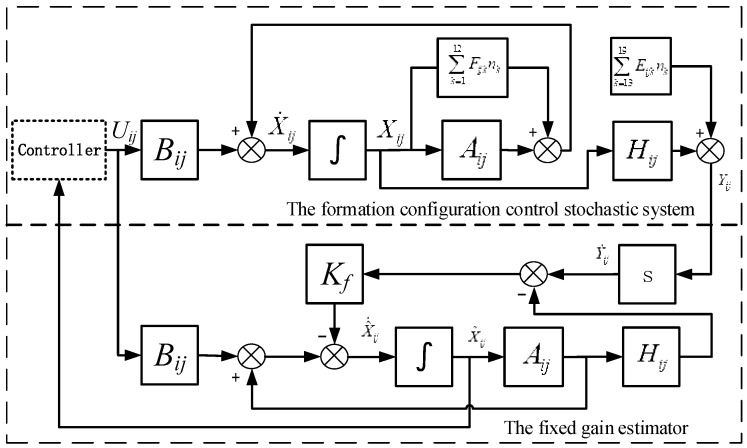
The framework of the closed-loop system.

In summary, the $It\hat{o}$ stochastic system of the USCC is as follows:

(49) $$\left\{ \begin{array}{l} dX_{ij}=A_{ij}X_{ij}dt+B_{ij}U_{ij}dt+\sum_{k=1}^{12}F_{ijk}X_{ij}dW_{k}\\ dY_{ij}=H_{ij}dX_{ij}+\sum_{k=13}^{19}E_{ijk}dW_{k}\\ U_{ij}=K_{c}K_{\omega ij}({\hat{X}}_{ij}-X_{ij}^{*})+U_{jd}\\ d{\hat{X}}_{ij}=(A_{ij}-K_{f}H_{ij}A_{ij}){\hat{X}}_{ij}dt+B_{ij}U_{ij}dt+K_{f}dY_{ij} \end{array}\right.$$

where the first equation is the state equation, the second is the measurement equation, the third is the control input, and the fourth is the state estimation equation. The above four equations together construct the expansion closed-loop equation for the stochastic system of USCC (as shown in Figure 2):

(50) $$d{\bar{X}}_{ij}=\left[{\bar{A}}_{ij}{\bar{X}}_{ij}+{\bar{B}}_{ij}(t)\right]dt+\sum_{k=1}^{19}\left[{\bar{F}}_{ijk}{\bar{X}}_{ij}+{\bar{G}}_{ijk}\right]dW_{i}$$

The states of the expansion system are:

(51) $${\bar{X}}_{ij}=\left[\begin{array}{c} X_{ij}\\ {\hat{X}}_{ij} \end{array}\right]$$

where ${\bar{X}}_{ij}\in R^{16\times 1}$; $X_{ij}\in R^{8\times 1}$ are original states; ${\hat{X}}_{ij}\in R^{8\times 1}$ are estimated states.

The state transfer matrix of the expansion system is:

(52) $${\bar{A}}_{ij}=\left[\begin{array}{cc} A_{ij} & B_{ij}K_{c}K_{\omega ij}\\ K_{f}H_{ij}A_{ij} & \left[(I_{8\times 8}-K_{f}H_{ij})A_{ij}+(I_{8\times 8}+K_{f}H_{ij})B_{ij}K_{c}K_{\omega ij}\right] \end{array}\right]$$

where ${\bar{A}}_{ij}\in R^{16\times 16}$; $A_{ij}\in R^{8\times 8}$ is the state transfer matrix of the original system; $B_{ij}\in R^{8\times 4}$ is the input matrix of the original system; $H_{ij}\in R^{7\times 8}$ is the estimate matrix of the original system; $K_{\omega ij}\in R^{8\times 8}$ is the adjacent adjustment matrix in $U_{ij}$; $K_{c}\in R^{4\times 8}$ is the control law; $K_{f}\in R^{8\times 7}$ is the gain of the estimator.

The input matrix of the expansion system is:

(53) $${\bar{B}}_{ij}(t)=\left[\begin{array}{c} -B_{ij}(K_{c}K_{\omega ij}X_{ij}^{*}-U_{jd})\\ -(I_{8\times 8}+K_{f}H_{ij})B_{ij}(K_{c}K_{\omega ij}X_{ij}^{*}-U_{jd}) \end{array}\right]$$

where ${\bar{B}}_{ij}(t)\in R^{16\times 1}$; $X_{ij}^{*}\in R^{8\times 1}$ is the system command; $U_{jd}\in R^{4\times 1}$ is the determined input of the adjacent node in $U_{ij}$. Note that: ${\bar{B}}_{ij}(t)$ is a time-varying matrix because $X_{ij}^{*}$ is time-varying.

The expansion stochastic state transfer matrix is:

(54) $${\bar{F}}_{ijk}=\left[\begin{array}{cc} F_{ijk} & 0\\ K_{f}H_{ij}F_{ijk} & 0 \end{array}\right]$$

where ${\bar{F}}_{ijk}\in R^{16\times 16}$; $F_{ijk}=0^{8\times 8}$(k=13~19).

The stochastic input matrix of the expansion system is:

(55) $${\bar{G}}_{ijk}=\left[\begin{array}{c} 0\\ K_{f}E_{ijk} \end{array}\right]$$

where ${\bar{G}}_{ijk}\in R^{16\times 1}$; $E_{ijk}=0^{7\times 1}$(k=1~12).

The standard Wiener process is:

(56) $$W={\left[W_{1},W_{2},\cdots ,W_{19}\right]}^{T}$$

where $W_{1}\sim W_{19}$ are the Wiener processes in (49); W is an independent 19-dimension standard Wiener process defined in the complete probability space: (Ω,F,P).

### 3.7. Main Results

Define the following matrix:

(57) $$M_{ij}={\bar{A}}_{ij}⊕{\bar{A}}_{ij}+\sum_{k=1}^{19}{\bar{F}}_{ijk}⊗{\bar{F}}_{ijk}$$

According to Theorem 1, the sufficient conditions of the stability of USCC stochastic model are:

(1) ${\bar{B}}_{ij}(t),{\bar{G}}_{ijk},k=1\sim 19$ are bounded.
(2) The real part of the eigenvalues of matrix $M_{ij}$ are negative.

It can be seen from (53) and (55) that all the elements in the matrices ${\bar{B}}_{ij}(t),{\bar{G}}_{ijk}$ are bounded according to their definition, because in $B_{ij}$, $K_{ip},T_{ip}$ are constant parameters of the thrust response and $K_{i\delta_{y}},T_{i\delta_{y}}$ are constant parameters of the elevator response; $K_{c}\in R^{4\times 8}$ is the control law which can be obtained after simulation; $K_{\omega ij}$ is an adjacency adjustment matrix whose elements are in the range of (0,1); $X_{ij}^{*}$ are bounded command values; $U_{jd}$ is the determined interference input vector of the adjacent node; $K_{f}\in R^{8\times 7}$ is the fixed gain of the estimator which can be determined from simulation; the nonzero elements in the matrix $H_{ij}\in R^{7\times 8}$ are ‘1’; and in ${\bar{G}}_{ijk}$, $E_{ijk}$(k=13~19) is the bounded variance vector of measuring noises. Therefore, we can further deduce the following:

**Proposition 1.**  *Under normal circumstances, a sufficient condition for the stability (or the mean square uniform boundedness of the stochastic system (50)) of the USCC system is that the real parts of the eigenvalues are negative, that is:*

(58) max{Reλ(Mij)}<0

*where the*max{Reλ(Mij)}*is the maximum real part of the eigenvalue of*$M_{ij}$.

## 4. Simulation and Experiments

With the aim of cooperative detection under complex environments, we ran simulations with two aircraft to verify the effectiveness of the proposed stochastic model. Moreover, the equivalent outfield flight test was carried out to complete the mission of cooperative detection in a certain area. The results demonstrate that the formation could be achieved effectively and accurately.

### 4.1. Simulation Results

#### 4.1.1. Initial State

To make it convenient for analysis, we set the number of UAV swarm n=2. The configuration of the formation during cruising is $x_{12}{}_{b}=100\;m$, $z_{12}{}_{b}=-173.2\;m$ (that is, $\nu_{2}$ located 100 meters forward and 173.2 meters left of $\nu_{1}$), and the cruising speeds are $V_{1b}=V_{2b}=100m/s$. The cruising trajectory declination is $\varphi_{1b}=\varphi_{2b}=0\;\mathrm{rad}$. The current configuration of the formation is the cruising formation, the current speed is $V_{1}=V_{2}=100\;m/s$, and the current flight path declination is $\varphi_{1}=\varphi_{2}=0\;\mathrm{rad}$. The original mass is $m_{1}=m_{2}=1400\;\mathrm{Kg}$, the y-components of inertial moments are $I_{y}{}_{1}=I_{y}{}_{2}=3980\;\mathrm{Kg}\cdot m^{2}$.

#### 4.1.2. Formation Parameters

Assume that the supporting network is strongly connected and that the adjacency coefficient $\omega_{ij}$ in (47) is 1.

#### 4.1.3. Standard deviation of random interference

Assume that the standard deviations of random interference in Equations (23) and (38) are:

$$\begin{array}{l} \sigma_{x_{ij}}^{}=1\;m,\;\sigma_{z_{ij}}^{}=1\;m,\;\sigma_{V_{i}}^{}=0.1\;m/s,\;\sigma_{P_{i}}^{}=5\;N,\;\sigma_{X_{i}{}^{V_{i}}}^{}=0.25\;\mathrm{kg}/s,\;\sigma_{Z_{i}{}^{V_{i}}}^{}=0.5\;\mathrm{kg}/s,\\ \sigma_{Z_{i}{}^{\beta_{i}}}^{}=0.5\;N/\mathrm{rad},\;\sigma_{Z_{i}{}^{\delta_{i}{}_{y}}}^{}=1.0\;N/\mathrm{rad},\;\sigma_{M_{iy}^{\beta_{i}}}^{}=1.0\;\mathrm{Nm}/\mathrm{rad},\;\sigma_{M_{iy}^{{\dot{\beta }}_{i}}}^{}=0.5\;\mathrm{Nms}/\mathrm{rad},\\ \sigma_{M_{iy}^{\omega_{iy}}}^{}=1.0\;\mathrm{Nms}/\mathrm{rad},\;\sigma_{M_{iy}^{\delta_{iy}}}^{}=1.0\;\mathrm{Nm}/\mathrm{rad},\;\sigma_{\Delta x_{ijm}}=4.8\;m,\;\sigma_{\Delta z_{ijm}}=4.8\;m,\\ \sigma_{\Delta v_{im}}=6.9\;m/s,\;\sigma_{\Delta \varphi_{im}}=0.005\;\mathrm{rad},\;\sigma_{\Delta \beta_{im}}=0.01\;\mathrm{rad},\;\sigma_{\Delta \omega_{iym}}=0.01\;\mathrm{rad}/s,\\ \sigma_{\Delta \delta_{iym}}=0.001\;\mathrm{rad}. \end{array}$$

#### 4.1.4. Formation Commands

The expected configuration of the system are $x_{ij}^{*}=50\;m$, $z_{ij}^{*}=-73.2\;m$, the expected formation speed is $V_{f}=100\;m/s$, and the expected formation declination is $\varphi_{f}=0\;\mathrm{rad}$.

#### 4.1.5. Optimal Design of Estimator Gain and Control Law

In order to improve the robustness of the stochastic system, the estimator gain in (43) and the control law in (45) are optimized by the SRAD [37].

**Figure 3:**
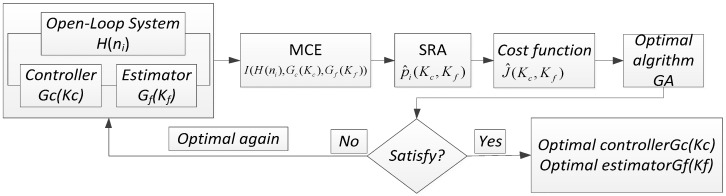
The design flow of SRAD.

The SRAD design flow is shown as in Figure 3, which is composed of two parts: a modern optimization algorithm and a control structure design. MCE denotes Monte Carlo evaluation, SRA denotes stochastic robustness analysis.

The cost function we designed is $J_{ij}=\sum_{i=1}^{12}w_{i}I_{i}{}^{2}(q_{i})$, where $q_{i}$ represents the 12 indicators which are shown in Table 1, $w_{i}$ is the weight of each indicator, and $I_{i}(\cdot )$ is the membership function of each indicator which obeys the rising-ridge distribution (59) or 0–1 distribution (60).

For indicators 1–9 and 12, the membership function obeys the rising-ridge distribution, i.e.,

(59) $$I(x)=\left\{ \begin{array}{cc} 0 & x\leq a\\ \frac{1}{2}+\frac{1}{2}\sin\frac{\pi }{b-a}(x-\frac{a+b}{2}) & a<x\leq b\\ 1 & x>b \end{array}\right.$$

where *a*, *b* are the best and allowable value of the indicators respectively. *x* is the simulation result.

For indicators 10 and 11, the membership function obeys the 0–1 distribution, i.e.,

(60) $$I(x)=\left\{ \begin{array}{ll} 1 & x<a\\ 0 & a\leq x\leq b\\ 1 & x>b \end{array}\right.$$

where (*a*, *b*) is the allowable range of the indicators, *x* is the simulation result.

${\hat{p}}_{i}(K_{c},K_{f})$ is the probability of indicator *i* that cannot satisfy the stability and requirements whose probability distribution function is $I_{i}(\cdot )$.

The minimum cost function J reflects the minimum probability that any indicator cannot satisfy the stability and requirements. It also indicates the minimum errors of the selected properties. Therefore, the obtained controller has high-quality robustness, and the probability that the control system does not meet the requirements is significantly reduced after multiple simulations.

The design steps in Figure 3 are as follows:

(1) Design the controller $G_{c}(K_{c})$ and estimator $G_{f}(K_{f})$ for the controlled object $H(n_{i})$;

(2) Define the indicators for SRAD: $I(H(n_{i}),G_{c}(K_{c}),G_{f}(K_{f}))$;

(3) Carry out Monte Carlo simulation on the closed-loop system to obtain the probability ${\hat{p}}_{i}(K_{c},K_{f})$ that cannot satisfy the stability and performance;

(4) Constitute a random cost function $\hat{J}(K_{c},K_{f})$ to satisfy both of robust stability and performance;

(5) Apply a modern optimization algorithm to get an optimal value. After we get the minimum value of $\hat{J}(K_{c},K_{f})$, then we obtain a stochastic robust optimal controller and an optimal estimator.

**Figure 4:**
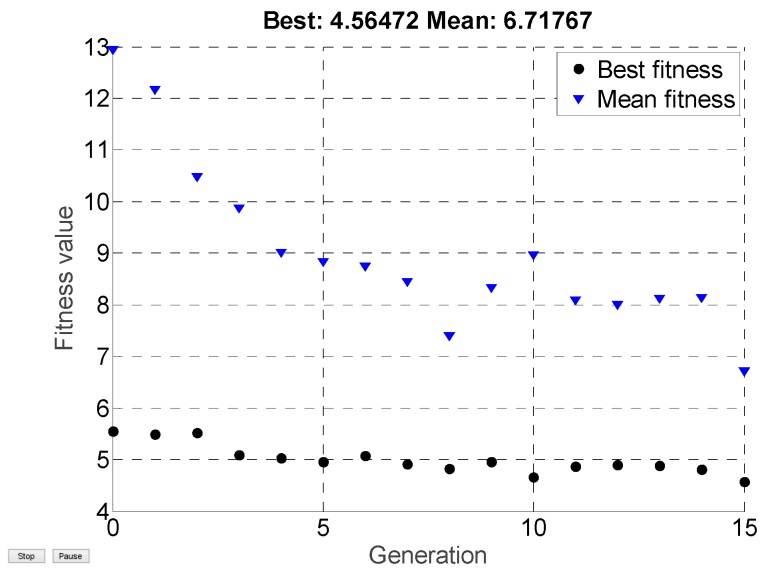
The iterative process of the genetic algorithm. (Note that the best fitness is the minimum value of the cost function in each iteration and the mean fitness is the mean value of the 100 Monte Carlo simulations in each iteration).

The optimization process of the designed parameters $K_{f}$ and $K_{c}$ is shown in Figure 4. After iterating 15 times with the genetic algorithm and running the Monte Carlo simulation 100 times per iteration, we got the optimal cost value: J=4.56 and the optimal parameters:

(61) $$K_{f}=\left[\begin{array}{ccccccc} 0.172 & & & & & & \\ 0 & 0.172 & & & & & \\ & 0 & 0.014 & & & & \\ & & 0 & 0.619 & & & \\ & & & 0 & 0.523 & & \\ & & & & 0 & 0.521 & \\ & & & & & 0 & 0\\ & & & & & & 0.796 \end{array}\right]$$

(62) $$K_{c}=\left[\begin{array}{cccccccc} 0.0408 & -0.0009 & -0.1157 & -0.0039 & 0.0164 & 0.0176 & 0 & 0.1894\\ 0.0089 & 0.0048 & -0.0274 & -0.8921 & 0.3046 & 0.3350 & 0 & -0.0012\\ 0 & & & \cdots & \cdots & & & 0\\ 0 & & & \cdots & \cdots & & & 0 \end{array}\right]$$

#### 4.1.6. Simulation Framework

**Figure 5:**
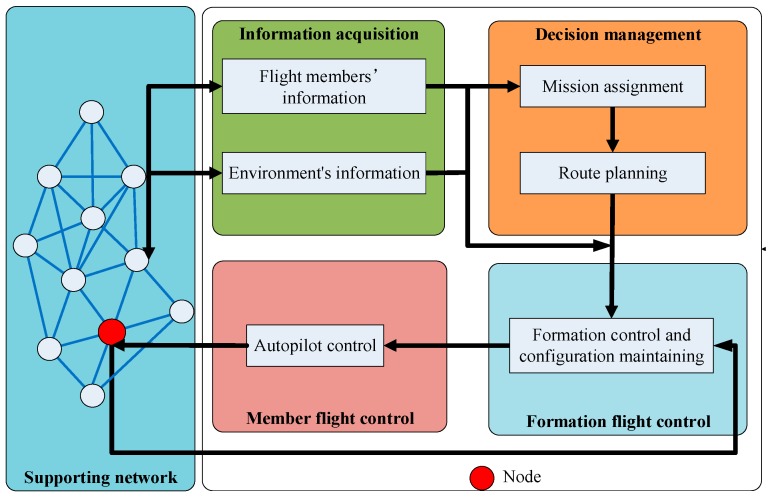
Simulation framework.

The framework of the simulation model is shown in Figure 5.

In the framework shown in Figure 5, the flight members in the formation are referred to nodes in the supporting network. The node obtains information through the formation support network and the sensor system, including neighbor nodes’ information and environment information. The decision management system allocates missions to flight members and plans the flight route for the formation. Finally, formation control system and member flight control system carry out missions using the $It\hat{o}$ stochastic system model (49) and the parameters presented above.

#### 4.1.7. Simulation Results

In order to better reflect the performance of the USCC stochastic system, we define the following indicator.

**Definition 2.**  *The weighted variance of the estimate error*${\tilde{X}}_{ij}=X_{ij}-{\hat{X}}_{ij}$*is:*

(63) $$V({\tilde{X}}_{ij})=\frac{1}{t_{f}-t_{0}}\int_{t_{0}}^{t_{f}}{\left({\tilde{X}}_{ij}-E\{{\tilde{X}}_{ij}\}\right)}^{T}W_{v}({\tilde{X}}_{ij}-E\{{\tilde{X}}_{ij}\})dt$$

where the diagonal matrix $W_{v}\in R^{8\times 8}$ is the weighted matrix of weighted variances, and the diagonal elements correspond to the states ${\hat{X}}_{ij}$ of the estimator. The larger the weight is, the more accurate the estimation of the corresponding state becomes. The trace of $W_{v}$ is $tr(W_{v})=1$. Assuming that the estimation accuracies of $\Delta {\hat{x}}_{ij}$, $\Delta {\hat{z}}_{ij}$, $\Delta {\hat{V}}_{i}$ and $\Delta {\hat{\varphi }}_{i}$ are required to be higher, the weighting matrix we designed is: $W_{v}=diag\left(0.2,0.2,0.2,0.2,0.1,0.05,0,0.05\right)$.

**Figure 6:**
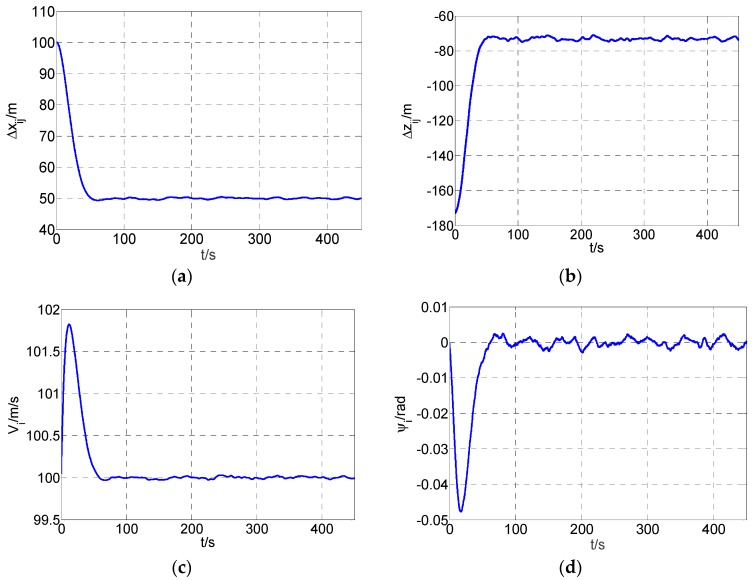
Simulation results of node ν_i._ (**a**) forward distance; (**b**) lateral distance; (**c**) speed; (**d**) flight path declination.

The simulation results correspond with the optimal cost value and the optimal parameters are shown as in Table 1 and Figure 6. Note that the fluctuation in the table refers to the standard deviation of the difference between the real-time configuration and the expected configuration.

It can be observed from Table 1 that all the indicators are in the appropriate range. The real part of the maximum eigenvalue is negative and the weighted variance of the estimation error calculated from simulation results is 3.86. From Figure 6 we can observe that the two aircraft achieved the desired configuration after 54.7 s, and the forward distance steady error is 0.0332 m, the lateral distance steady error is 0.0944 m. The formation speed and the formation declination meet the designed requirements well. Thus the system is mean-square uniform bounded according to Proposition 1.

### 4.2. Autonomous Flight Experiments

**Figure 7:**
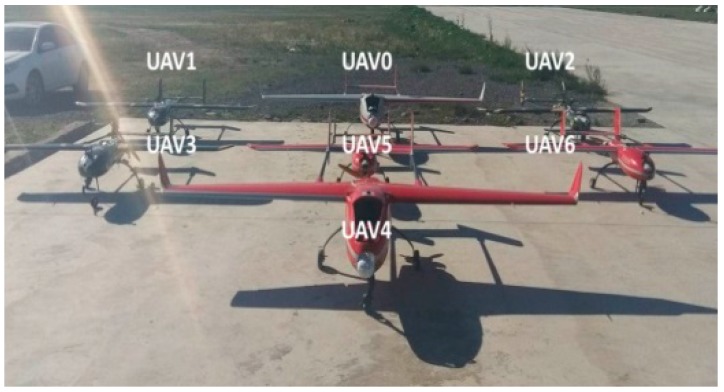
Seven UAVs.

In order to observe the performance of the model and verify the effectiveness of the USCC stochastic system, we conducted an equivalent outfield autonomous formation flight test by using multiple UAVs. As shown in Figure 7, we carried out experiments with seven nonholonomic UAVs. The UAV swarm can cooperatively search the certain area with different configurations.

**Figure 8:**
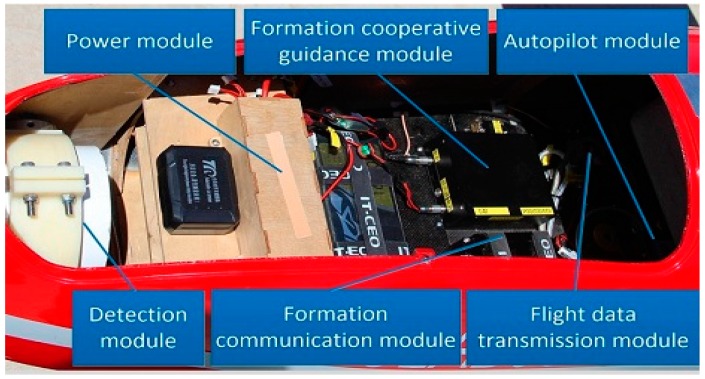
The loads in the cabin of the UAV.

**Figure 9:**
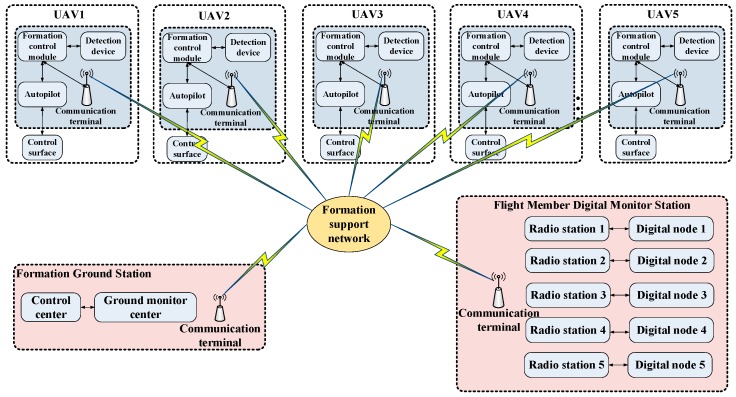
The framework of the USCC outfield flight system.

The loads in each cabin of the UAV are shown in Figure 8, including the power module, formation cooperative guidance module, autopilot module, detection module, formation communication module and flight data transmission module. In the experiment, we adopted the proposed USCC stochastic model into the formation cooperative guidance module to instruct the flight members to reach the desired position and maintain a steady configuration. The framework of the whole system is shown in Figure 9.

The formation ground station is set to observe the real-time formation flight process, and upload commands to instruct the formation to change its configuration or adjust relative distances. It can also control the pod to capture the configuration. The flight member’s digital monitor station is set to observe the real-time flight member’s flight statuses and to ensure the safety of the whole flight process.

**Figure 10:**
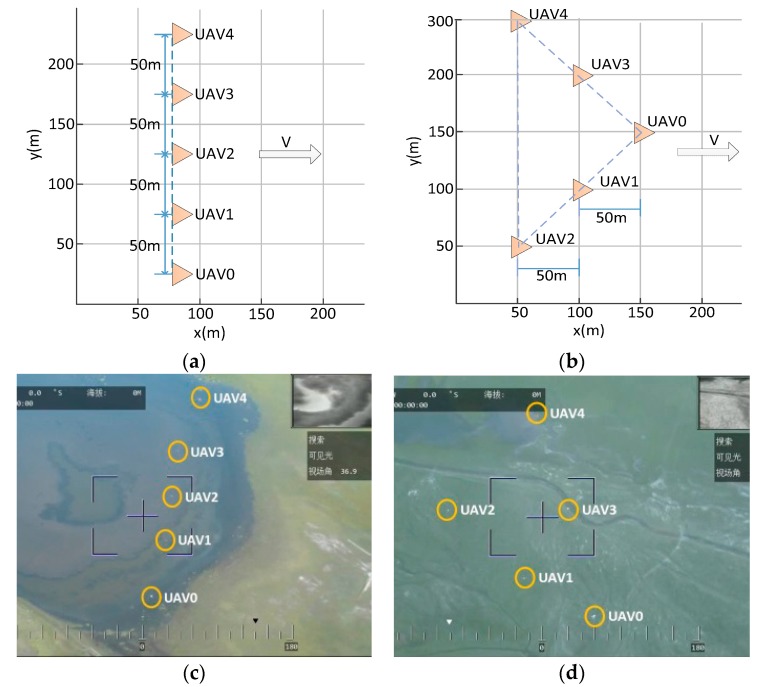
Configurations of five drones. (**a**) Designed lateral configuration; (**b**) Designed wedge configuration; (**c**) Real time lateral configuration; (**d**) Real time wedge configuration.

Two configurations of five drones we designed in the experiments are shown in Figure 10a,b. The lateral and forward distance between neighbor UAVs was set to 50 m. Moreover, the configurations in Figure 10c,d were captured by the UAV that was flying higher with a pod.

It can be observed from Figure 10 that the UAVs achieved the desired configuration smoothly and maintained the formation effectively under the proposed model.

**Figure 11:**
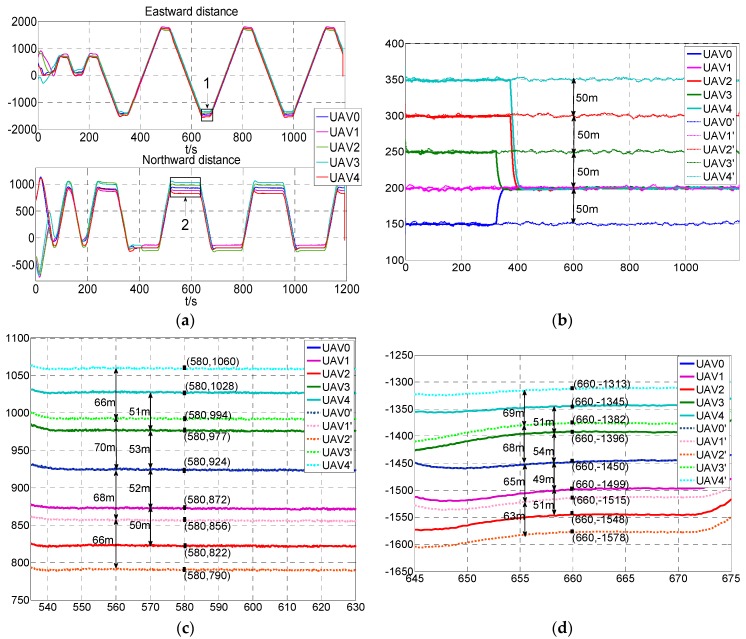
Experimental results of the wedge configuration. (**a**). The flight path of the five UAVs; (**b**). The height of five UAVs. The flight paths in full line belong to the UAV0 to UAV4 with USCC stochastic system model while the dotted line belong to the UAV0’ to UAV4’ are without the model; (**c**). Northward distances between the five UAVs which are indicated by “2” in (**a**). The flight paths in full line of UAV0 to UAV4 utilize the USCC stochastic system model while the dotted line of UAV0’ to UAV4’ do not use the model; (**d**). Eastward distances between the five UAVs which are indicated by “1” in (**a**). The flight paths in full line of UAV0 to UAV4 use USCC stochastic system model while the dotted line of UAV0’ to UAV4’ do not use the model.

For the convenience of analysis, the actual flight data could be saved through the formation monitoring station. In this paper, we take the flight data of the wedge configuration with and without the USCC stochastic system model in the experiment to invert the flight process and evaluate the effectiveness of the USCC stochastic system. The results are shown in Figure 11.

The curves shown as in Figure 11 demonstrate that the five UAVs achieved the desired configuration and steadily maintained the formation. The flight paths in the rectangle that the arrows “1” and “2” point to in Figure 11a are amplified in Figure 11c,d. The data are summarized in Table 2, from which we can observe that in the flight test not using the model, the relative height of the UAVs should be maintained at the very least at 50 m to avoid the risk of collision, while the heights of the five UAVs with USCC stochastic system model converged to 200 m and the relative distance in height was zero. The average 3D distance between flight members in the flight test with the proposed model was shortened by 32.14% compared to the test without the model.

The results show that the introduction of multiplicative noises improves the formation maintenance performance and extends the boundary properties of the formation flight, such as the safety distance is shorter and the relative height can be eliminated. Therefore, the proposed stochastic model could provide the UAV swarm with a larger maneuvering space, and then improve the efficiency and quality of mission execution, enhance the operational capability in high-risk environments and improve the adaptation of the system to the complex environment.

## 5. Conclusions

In this paper, the problem of the state estimation and control of the UAV swarm system with the consideration of multiplicative noises is studied. The closed-loop $It\hat{o}$ stochastic system we constructed is the combination of a state equation introduced from group kinematic model and individual dynamic model considering the multiplicative noises, an observation equation considers the measurement noises, an estimator and a controller. Following that, the proof to verify the mean-square uniform boundedness of the system is presented. The optimal estimator and controller are obtained with the use of SRAD in the simulation. Finally, simulation results show that the system is stable and the selected indicators meet the requirements. The outfield experiment results demonstrate that the configuration with the proposed model could be significantly condensed by 32.14% compared to the test with the traditional model. Therefore, the stochastic system of USCC with multiplicative noises proposed in this paper could contribute to effectively exploiting the boundary performances of the system and constructing a high dynamic formation in practical application, thus better matching the actual environment.

However, there is much to be researched further in this area. For example, in the practical application, the time delay cannot be ignored, especially for large scale formations. The modeling of the USCC stochastic system considering time delay is currently under investigation.

## Figures and Tables

**Table 1:** The stability and indicators.

Indicators	Weight	Membership (a,b)	Results
1.Stability (the real part of the maximum eigenvalue is negative)	10	(−0.0001, 0)	−0.00001
2. Forward distance adjust time	1	(0, 75 s)	54.7 s
3. Lateral distance adjust time	1	(0, 75 s)	49.8 s
4. Forward distance overshoot	5	(0, 10%)	1.42%
5. Lateral distance overshoot	5	(0, 10%)	2.51%
6. Forward distance steady error	1	(0, 1 m)	0.0332 m
7. Lateral distance steady error	1	(0, 1 m)	0.0944 m
8. Forward distance fluctuation	1	(0, 2 m)	0.2586 m
9. Lateral distance fluctuation	1	(0, 2 m)	0.9057 m
10. Average velocity instruct	3	(90 m/s, 110 m/s)	100.07 m/s
11. Average flight path declination instruct	3	(−1.57 rad, 1.57 rad)	0.0644 rad
12. The weighted variance of the estimation error	2	(0, 10.0)	3.86

**Table 2:** The data of configurations with and without USCC stochastic system model.

Indicators	Configuration Data of Proposed Model	Original Configuration Data
Eastward average distance	51.25 m	66.25 m
Northward average distance	51.5 m	67.5 m
Average relative height	0 m	50 m
Average 3D relative distance	72.6 m	107.0 m
